# Neem as a Natural Preservative in Postmortem Care: A Case Report

**DOI:** 10.7759/cureus.88888

**Published:** 2025-07-28

**Authors:** Treasa James, Akhilesh Pathak

**Affiliations:** 1 Forensic Medicine, All India Institute of Medical Sciences, Bathinda, Bathinda, IND

**Keywords:** autopsy, drowning, embalming, neem, postmortem, preservation

## Abstract

This report explores the potential role of neem (*Azadirachta indica*) as a natural postmortem preservative. A 33-year-old male who died due to drowning was brought for autopsy several days after death, covered entirely in neem leaves by his family. Despite the delay, decomposition was notably less advanced than typically expected, with reduced skin slippage, bloating, and putrefactive odor. This observation suggests neem’s antimicrobial and antifungal properties may help delay postmortem changes. In resource-limited or culturally specific settings, neem may offer a temporary, plant-based alternative to traditional preservation methods, warranting further scientific investigation.

## Introduction

Preserving the human body after death is a practice that blends science, tradition, and evolving technology. It can be done naturally through freezing, drying, using specific soil types, or artificially using methods such as embalming. While ancient cultures relied on substances such as natron, resins, or honey, modern embalming typically involves injecting preservative chemicals into arteries and body cavities to slow decomposition. Concerns over the health risks and environmental impact of traditional chemicals, especially formaldehyde, have prompted the development of safer alternatives such as glycerine, thymol, and natural oils. Advances now focus on preserving the body’s appearance, flexibility, and tissue integrity for teaching and research purposes, balancing anatomical utility with sustainability and safety [1].

Modern embalming primarily involves arterial injection and cavity treatment to prevent microbial growth and decomposition, especially in settings involving education, research, or transportation of remains. While formaldehyde remains widely used, often in combination with ethanol, phenol, and glycerine, its toxicity has led to the integration of humectants, pH buffers, and dyes to improve both preservation and appearance. Innovative methods such as the Thiel technique and soft embalming with alcohol and salts have further enhanced tissue flexibility and realism for surgical training [2].

Embalming continues to evolve, balancing anatomical utility with safety and environmental concerns. The method chosen depends on the body’s intended use, whether for education, research, funeral preparation, or long-term display. With increasing awareness of the health risks posed by traditional chemicals, ongoing research seeks alternative, safe, and effective formulations [1-3].

Neem (*Azadirachta indica*), a tree deeply rooted in traditional medicine across the Indian subcontinent, is renowned for its potent antimicrobial, antifungal, and insect-repelling properties. Its key bioactive compounds, nimbin, nimbidin, and azadirachtin, have effectively inhibited bacterial and fungal growth [4,5]. While neem is widely known for its applications in health and agriculture, it also holds promise in postmortem care. By discouraging microbial activity and deterring insects, neem helps create a more stable environment that can delay visible decomposition and reduce unpleasant odors. This offers a practical, culturally appropriate solution in forensic contexts where refrigeration or chemical embalming is unavailable [6,7].

While modern embalming has evolved significantly, it still relies heavily on chemical agents that pose health and environmental risks. At the same time, many regions continue to face practical challenges, such as a lack of refrigeration or access to embalming facilities, making temporary preservation of the deceased a pressing concern. Surprisingly, despite neem’s powerful antimicrobial and insect-repellent properties and its deep roots in traditional Indian practices, there is little to no published research on its use in preserving human bodies after death. This gap is particularly striking given the need for accessible, culturally relevant, and safe alternatives in low-resource settings. This report presents the first documented use of neem leaves for temporary human body preservation, offering insight into its practical application and potential as a natural, low-cost solution.

## Case presentation

**Figure 1:**
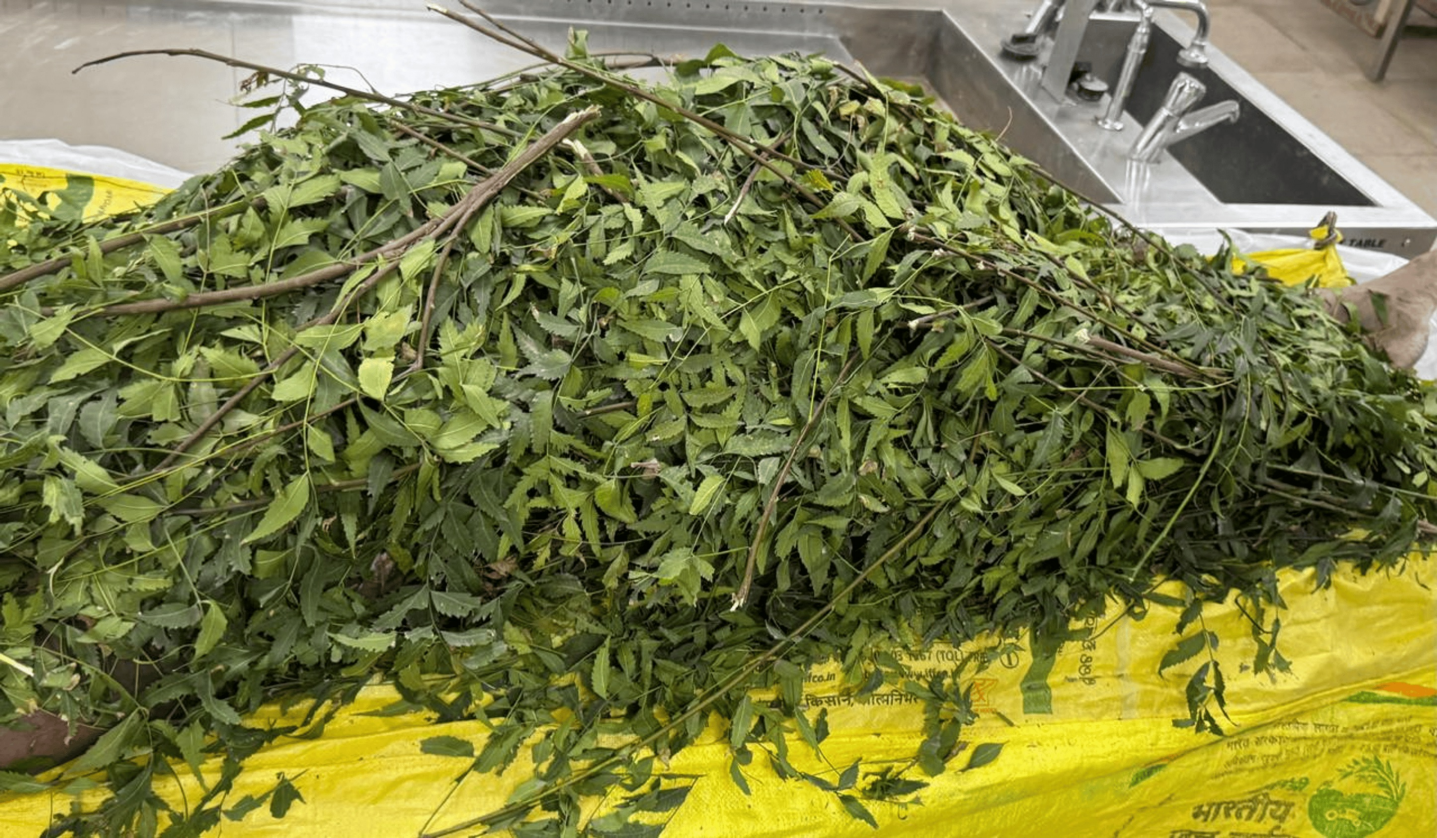
The body of the deceased covered with neem leaves.

A 33-year-old male was brought for postmortem examination following a suspected case of drowning. As per available police records, the deceased was last seen alive two days before the recovery of the body. The body was discovered floating in a local water body by passersby and was subsequently recovered by authorities. Due to procedural delays in conducting the inquest and transporting the body, the autopsy was performed five days after recovery. What made this case particularly unusual was the condition in which the body was received: completely covered with neem (*Azadirachta indica*) leaves by the relatives, as shown in Figure 1.

The family used the leaves as a traditional method of preservation, believing it would slow decomposition and reduce odor until the autopsy could be conducted.

On external examination, the body showed early signs of decomposition, such as mild abdominal bloating, greenish discoloration of the lower abdomen, and skin slippage over dependent areas. Interestingly, despite the prolonged postmortem interval, the extent of external putrefaction was less pronounced than typically observed. The characteristic putrefactive odor was also markedly subdued. This attenuation of external decomposition may be attributed to the use of neem leaves, known for their antimicrobial and insect-repelling properties.

**Figure 2:**
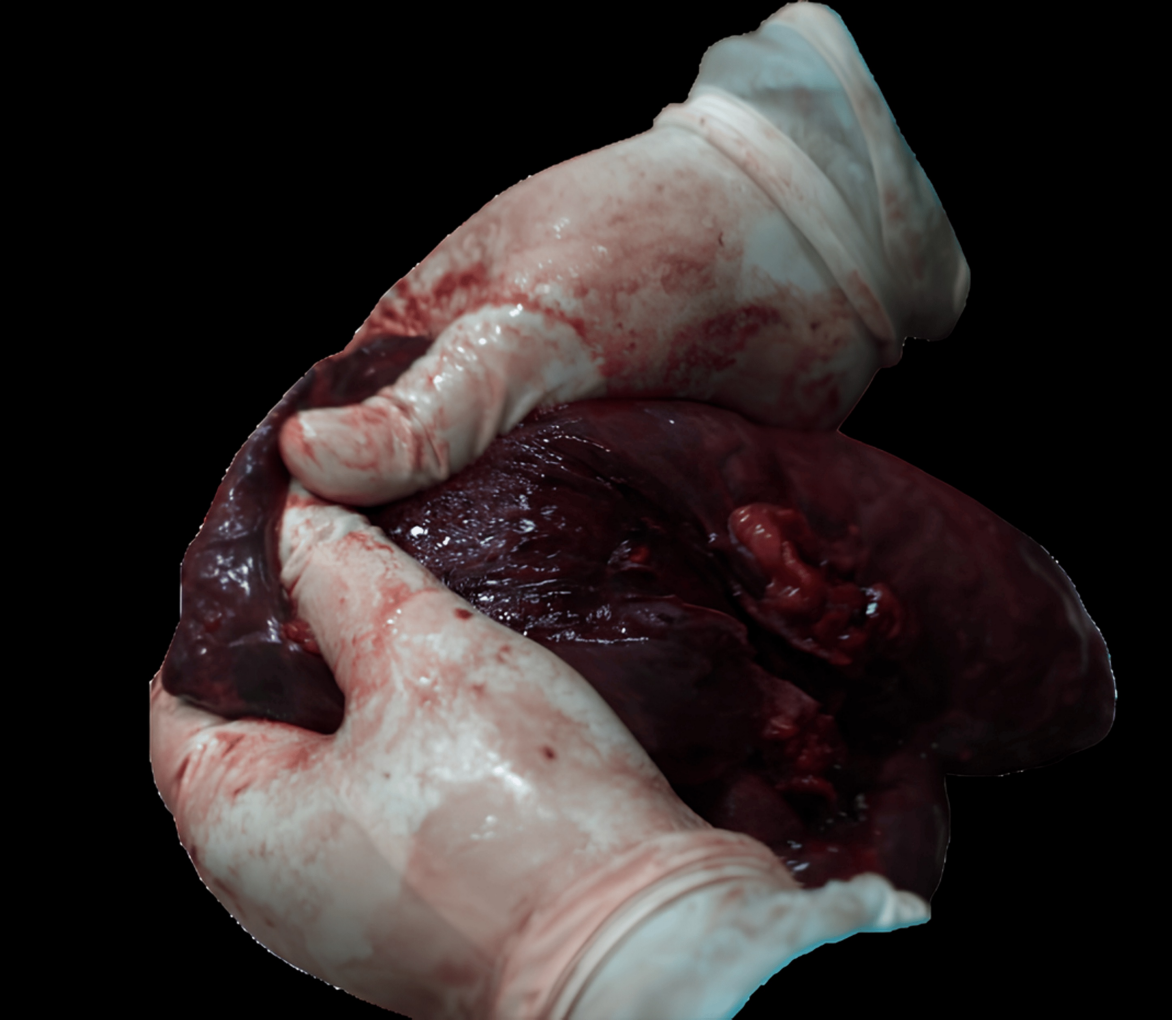
Cut section of the lungs showing deep congestion and the presence of frothy white discharge on compression, a classical finding suggestive of death due to drowning.

On internal examination, morphological changes consistent with a postmortem interval of approximately five days were observed, including generalized organ softening, discoloration due to hemolysis, and early liquefaction, particularly in the lungs. These changes are characteristic of internal decomposition following death due to drowning, where moist environments accelerate autolytic and putrefactive processes. Notably, a cut section of the lungs revealed frothy white discharge (Figure 2), a classical feature suggestive of death due to drowning.

No external or internal injuries were noted on thorough examination, and no signs of foul play were detected. The stomach contained watery fluid without any unusual odor or content. Viscera were preserved and sent for chemical analysis, including the stomach contents, liver, kidney, and blood, to rule out any possible toxicological cause. The chemical analysis report returned negative for common poisons, confirming no toxic contribution to death. Despite the external application of *Azadirachta indica* (neem) leaves, which may have delayed superficial decomposition, internal findings progressed as expected, indicating that neem’s preservative effects were limited to surface-level tissues. However, the degree of putrefaction was relatively moderate compared to expected findings for the time elapsed.

This case underscores a fascinating intersection between indigenous knowledge and forensic medicine. While neem leaves cannot replace modern preservation techniques, such as refrigeration or embalming, their use in this case appeared to have delayed external decomposition and suppressed the odor, offering temporary benefits in settings lacking advanced mortuary infrastructure. The active constituents of neem, such as nimbin, nimbidin, and azadirachtin, may have inhibited microbial activity on the body surface. However, internal decomposition proceeded in line with expectations, indicating the limited depth of neem’s preservative effect.

This observation suggests potential for plant-based interventions in postmortem care in resource-limited settings. While the gross features observed in this case suggest a potential preservative effect of neem leaves on external decomposition, further research is required to scientifically validate these findings, as no histological or biochemical analyses were performed to confirm the observations.

## Discussion

Over the centuries, various methods have been used to preserve human bodies, primarily to delay decomposition for anatomical study, transportation, or funeral rites. Batra et al. [2] described embalming as both an art and a science, relying on germicidal and preservative chemicals introduced into the vascular system and body cavities to prevent putrefaction and maintain a visually acceptable appearance. Ancient civilizations such as the Egyptians used natron and spices for mummification, while modern embalming has evolved to include arterial, cavity, hypodermic, and surface techniques using chemicals such as formaldehyde, glutaraldehyde, and phenol. Although these methods are highly effective, they may complicate medicolegal investigations by altering tissue structures and potentially masking signs of trauma or toxins. In areas with limited access to embalming facilities, traditional alternatives, such as neem (*Azadirachta indica*) leaves, have been employed for their natural antimicrobial and insect-repelling properties, offering short-term preservation benefits. However, such indigenous practices are rarely documented in scientific literature. What sets our case apart is that it captures, within a forensic context, the real-time application of neem leaves by the deceased’s family to preserve the body during an unavoidable delay. This highlights not only the practical impact of a culturally rooted method but also its visible effect on slowing external decomposition. This observation warrants further scientific exploration.

Brenner’s 2014 review [1] provided a comprehensive overview of the evolution of human body preservation, from ancient mummification practices in Egypt and the Chinchorro culture, driven by spiritual beliefs and environmental needs, to the innovations of the Renaissance by figures such as Frederik Ruysch and Leonardo da Vinci, who introduced early arterial injection techniques. Today, anatomical embalming largely depends on formaldehyde due to its potent preservative effects. However, growing concerns about health risks, including carcinogenicity, have prompted the search for safer alternatives that can preserve tissue flexibility and maintain a lifelike appearance. Brenner’s review also emphasizes the influence of regulatory frameworks such as the European Biocidal Products Directive in shaping the future of embalming practices. While Brenner focuses on chemical and policy-driven advancements, our case demonstrates a grassroots, culturally embedded method using neem leaves, highlighting its practical relevance in rural settings where conventional preservation methods are unavailable.

A 2023 pilot study by Rathore et al. [8] investigated a novel herbal alternative to formalin for preserving human muscle tissue, aiming to address the toxicity and irritant effects associated with traditional preservatives. The team developed a solution using neem, karanja, citric acid, and alcohols through maceration. Over a two-month period, they evaluated tissue consistency, odor, and structural integrity, finding that the 100% herbal formulation maintained preservation without decay or insect activity. These findings suggest a promising eco-friendly alternative to formalin with potential anatomical applications. In contrast to this controlled laboratory setting, our case report documents the real-world use of neem leaves alone in a forensic context to delay decomposition, reinforcing their practical utility during autopsy delays in resource-limited environments.

Alsharif et al. [3] offered an extensive review of cadaver preservation methods, focusing on how microbial activity, primarily from bacteria and fungi, drives decomposition, and how various preservation techniques aim to counteract it. They discussed both traditional approaches, such as natural mummification via environmental extremes, and modern innovations such as plastination, which uses polymers such as silicone, epoxy, or polyester to create odorless, durable, and visually accessible specimens. The review also compared the strengths and limitations of embalming techniques, such as arterial, cavity, hypodermic, and surface, and emphasized the need for ongoing microbial surveillance to preserve cadaveric quality. While the review by Alsharif et al. centered on sophisticated laboratory-based methods, our case report brings attention to a practical, culturally informed technique using neem leaves to preserve an unembalmed body in real-world forensic conditions.

In their review, Protano et al. [9] evaluated the carcinogenic risks associated with formaldehyde exposure, particularly in occupational settings. Drawing from epidemiological studies, they reported strong links between formaldehyde exposure and nasopharyngeal cancer, leukemia, especially myeloid leukemia, and other upper respiratory tract malignancies. The International Agency for Research on Cancer classifies formaldehyde as a Group 1 human carcinogen, underscoring the health risks for professionals working in embalming rooms and anatomy laboratories. Their findings highlight the ongoing dilemma between effective preservation and occupational safety. Although formalin remains the gold standard for cadaver fixation due to its strong preservative properties, its toxic effects, including mucosal irritation, respiratory distress, and carcinogenic potential, necessitate the development of safer alternatives. This aligns with global trends favoring less hazardous methods in medical and forensic practice. Our case report complements this concern by presenting neem leaves as a natural, non-toxic, and accessible option for short-term external preservation during delays, especially in rural settings where refrigeration and embalming are not feasible.

Drowning remains a diagnostically challenging form of asphyxial death due to the absence of pathognomonic findings. Traditional autopsy indicators such as frothy fluid in the airways, pulmonary edema, and water in the stomach can aid diagnosis, but often overlap with findings from other causes of death. To improve diagnostic accuracy, Nagar et al. [10] proposed the Drowning Index (DI), calculated as the ratio of combined lung and pleural effusion weight to spleen weight. Their study demonstrated that a DI >9.7 had a sensitivity of 86.7% and specificity of 70.2% for diagnosing freshwater drowning within a postmortem interval of up to two weeks. This index outperformed lung weight alone and proved especially valuable in decomposed bodies, where classical signs may be diminished. The authors also emphasized that pleural effusion accumulation increases with time and environmental factors, while spleen weight typically decreases in drowning. In the present case, although biochemical indices such as DI were not measured, the presence of frothy airway secretions, heavy edematous lungs, and absence of alternative fatal pathology supported the diagnosis of freshwater drowning. The gross anatomical features correlated with the inference of drowning despite the delayed autopsy and absence of advanced diagnostic tools.

While this case highlights the potential of *Azadirachta indica* (neem) as a natural postmortem preservative, the findings remain observational and anecdotal. No biochemical, microbial, or histopathological analysis was performed to objectively validate the effect of neem. Nonetheless, in an era where environmental toxicity is rising due to excessive chemical use, revisiting traditional, eco-friendly methods may offer sustainable alternatives. This report underscores the need for future controlled studies to evaluate and standardize the use of plant-based agents in postmortem care.

## Conclusions

This case prompts a re-evaluation of postmortem preservation practices through the integration of traditional knowledge and modern forensic needs. Neem (*Azadirachta indica*), long valued in indigenous medicine for its antimicrobial and insect-repelling properties, demonstrated a visible delay in external decomposition and a notable reduction in putrefactive odor when applied in a real-world setting. In the absence of refrigeration or chemical embalming, this culturally rooted method offered temporary preservation, creating not only a more dignified condition for the deceased but also a safer and more tolerable environment for forensic personnel during autopsy. Importantly, these effects were appreciated solely through gross examination, without histological or microbiological confirmation. This observation raises important questions about the potential of plant-based preservation methods as accessible, ethical, and sustainable adjuncts to conventional techniques. While neem cannot replace formalin-based preservation, its application in this context highlights the need for systematic, controlled research to validate its efficacy and explore its possible integration into standardized forensic protocols. As forensic medicine continues to evolve, such intersections of tradition and evidence-based practice warrant deeper exploration and could inspire novel, context-sensitive approaches to postmortem care.
